# High mortality and amphotericin B-related nephrotoxicity in cerebral cryptococcosis in a low-resource neurology referral center: a prospective cohort study

**DOI:** 10.3389/fneur.2026.1776988

**Published:** 2026-03-09

**Authors:** Johanne Yorshire Lobo Requena, Máximo Quintero, Freddy Pachano Arenas, Vanessa Belloso de Noriega, Yadira Marin-Hamburger, Sulaiman Kalokoh, Fabriccio J. Visconti-Lopez

**Affiliations:** 1 Universidad del Zulia, Maracaibo, Venezuela; 2 Universidad de la Costa, Barranquilla, Colombia; 3 Faculty of Information and Communication Technology, Limkokwing University of Creative Technology, Freetown, Sierra Leone; 4 Universidad Científica Del Sur, Lima, Peru

**Keywords:** acute kidney injury, amphotericin B, cryptococcosis, *Cryptococcus neoformans*, HIV infections

## Abstract

**Background:**

Cerebral cryptococcosis (neurocryptococcosis) is an important cause of meningoencephalitis among immunocompromised people worldwide, particularly in regions with limited access to antiretroviral therapy and to optimal antifungal agents.

**Methods:**

Prospective observational cohort study of consecutive adults hospitalized with cerebral cryptococcosis at the Neurology Service of the Servicio Autónomo Hospital Universitario de Maracaibo, Maracaibo, Zulia, Venezuela (January 2022 to July 2024). Data were collected using a validated instrument; modified Rankin Scale (mRS) assessed disability at discharge.

**Results:**

Sixty-two patients (mean age 36.5 ± 12.9 years; 66.1% male) were included; 72.6% were HIV-positive. All patients presented headache, neck stiffness and altered mental status. Mean CSF protein was 103.5 mg/dL, cell count 504.5 cells/mm^3^ and glucose 33.8 mg/dL. Amphotericin B (daily regimen) was administered in 74.2% and fluconazole in 82.3%. Acute kidney injury (AKI) occurred in 59.7% and hypokalemia in 56.5%. In-hospital mortality was 46.8% (mRS 6). Mortality was higher in patients with AKI (35.5% vs. 11.3%; *p* = 0.015) and hypokalemia (*p* = 0.017). Mortality tended to be lower with daily versus alternate-day amphotericin (10% vs. 45%; *p* = 0.297).

**Conclusion:**

Cerebral cryptococcosis in this referral setting affects predominantly young, HIV-infected adults and is associated with high mortality and disability. Amphotericin B-related nephrotoxicity and hypokalemia were common and associated with worse outcomes, underscoring the need for nephroprotection protocols and optimized antifungal strategies in resource-limited settings.

## Introduction

Cerebral cryptococcosis (neurocryptococcosis) is an important cause of meningoencephalitis among immunocompromised people worldwide, particularly in regions with limited access to antiretroviral therapy and optimal antifungal agents (1, 2). The infection produces substantial intracranial morbidity through meningeal inflammation, raised intracranial pressure and parenchymal involvement; outcomes are further complicated by toxicities of induction therapy, especially amphotericin B–associated nephrotoxicity and electrolyte abnormalities (3). Although global guidelines recommend amphotericin B plus flucytosine for induction, flucytosine and lipid amphotericin formulations are frequently unavailable in low- and middle-income countries, where amphotericin B deoxycholate and fluconazole remain the main therapeutic options (4). Local, contemporaneous clinical series are needed to document presentation patterns, laboratory profiles, treatment practices and the frequency of treatment-related complications that could be amenable to targeted mitigation (4). This prospective study aimed to describe the demographic and clinical characteristics, CSF findings, antifungal regimens, adverse events and short-term outcomes of patients with cerebral cryptococcosis admitted to a tertiary neurology referral service in Maracaibo, Venezuela, and to explore associations between treatment practices, complications and in-hospital outcomes.

## Methods

### Study design and setting

Prospective observational cohort study of consecutive adults hospitalized with cerebral cryptococcosis at the Neurology Service of the Servicio Autónomo Hospital Universitario de Maracaibo (SAHUM), Maracaibo, Zulia, Venezuela (January 2022 to July 2024).

### Participants and sampling

All consecutive adult patients hospitalized with a diagnosis of cerebral cryptococcosis who provided informed consent were enrolled. Exclusion criteria were severe cognitive impairment or dementia precluding consent, pregnancy, preexisting chronic kidney disease and receipt of antimicrobials for <3 days. Sampling was non-probabilistic consecutive (all eligible admissions during the study period).

### Data collection and definitions

A standardized instrument, developed by the investigators and validated by two clinical experts, captured demographics (age, sex, residence), presenting neurological signs and symptoms, CSF parameters (protein, glucose, total cell count, India ink), imaging findings when available, antifungal regimens (amphotericin B deoxycholate schedule and cumulative dose) and adjunctive antimicrobials, length of hospitalization, adverse events, and the modified Rankin Scale (mRS) score at discharge. Cumulative amphotericin B deoxycholate dose (mg) was defined as the total amount administered during induction therapy as documented in the medical record; because patient weight and dose-escalation details were not uniformly recorded, dosing could not be standardized as mg/kg/day or summarized as escalation patterns for all patients. Acute kidney injury (AKI) was recorded as a chart-documented clinical event during hospitalization based on renal-function assessment available in routine care; serial serum creatinine and derived indices (e.g., BUN and eGFR) were not consistently documented for all patients, so AKI staging was not feasible. Hypokalemia was defined as any serum potassium value below the institutional lower limit of normal recorded during hospitalization; pre-admission potassium values were not consistently available to classify chronic versus treatment-emergent hypokalemia.

### Diagnostic approach and inclusion of India-ink negative cases

Diagnosis reflected routine clinical practice in a low-resource setting and was based on a compatible meningoencephalitis syndrome with supportive CSF findings and mycological testing when available. India-ink microscopy was performed as part of standard care. For patients with negative India-ink staining, cases were included when the treating neurology team documented a diagnosis of cerebral cryptococcosis and initiated antifungal therapy; when available, cryptococcal antigen testing (CrAg) and/or CSF fungal culture supported the diagnosis. Because CrAg testing and CSF culture were not consistently available during the study period, not all India-ink negative cases had confirmatory microbiologic documentation, and these cases should be interpreted as clinically diagnosed under diagnostic constraints. Follow-up was limited to the hospitalization episode; post-discharge outcomes and longer-term functional status were not systematically collected.

### Statistical analysis

Analyses used SPSS v24. Continuous variables are reported as mean ± SD; categorical variables as counts and percentages. Differences between groups were evaluated with Student’s *t*-test for continuous variables and chi-square test or Fisher’s exact test for categorical variables. A two-sided *p* < 0.05 denoted statistical significance. Associations of interest included treatment schedule (daily vs. alternate-day amphotericin), presence of AKI and hypokalemia, and outcomes (in-hospital mortality and mRS at discharge).

### Ethics

The study was approved by the postgraduate coordinator and the head of the Neurology Service at SAHUM. All participants or surrogates provided written informed consent prior to inclusion.

## Results

Sixty-two patients were included. Baseline characteristics and outcomes are summarized in Table 1. The cohort had a mean age of 36.5 ± 12.9 years (range 15–63); 66.1% were male and 79.0% originated from rural areas. HIV infection was documented in 72.6% (*n* = 45) of patients. All patients presented with headache, neck stiffness and altered mental status. Additional neurologic findings included motor deficit in 56.5% and seizures in 22.6%; cranial nerve involvement most frequently included supranuclear VII palsy (19.3%).

**Table 1:** Key baseline characteristics and outcomes.

Variable	Result (*n* = 62)
Mean age, years (SD)	36.5 ± 12.9
Male sex, *n* (%)	41 (66.1%)
HIV positive, *n* (%)	45 (72.6%)
Mean hospital stay, days	14.95
Amphotericin B deoxycholate schedule (daily), *n* (%)	46 (74.2%)
Fluconazole, *n* (%)	51 (82.3%)
Acute kidney injury, *n* (%)	37 (59.7%)
Hypokalemia, *n* (%)	35 (56.5%)
In-hospital mortality (mRS 6), *n* (%)	29 (46.8%)
Mean CSF protein, mg/dL	103.5
Mean CSF cell count, cells/mm^3^	504.5

CSF analysis revealed mean protein 103.5 mg/dL, mean cell count 504.5 cells/mm^3^ with lymphocytic predominance (98% mononuclear), and mean glucose 33.8 mg/dL. India-ink microscopy was negative in 46.8% of cases, underscoring its limited sensitivity in this series. Because cryptococcal antigen testing and CSF culture were not consistently available, microbiologic confirmation was not possible for all cases. As a result, some India-ink negative cases may have been included on the basis of a clinician-documented diagnosis with supportive CSF findings in the absence of CrAg/culture confirmation. Elevated CSF adenosine deaminase (ADA) was noted in 11.3% and prompted consideration of coinfection with *Mycobacterium tuberculosis* in selected cases.

Treatment practices reflected resource constraints: amphotericin B deoxycholate was used as induction in 74.2% on a daily schedule and in 22.6% on an alternate-day schedule; 82.3% received fluconazole as part of induction/maintenance due to lack of flucytosine availability. Among patients who received amphotericin B deoxycholate, the mean cumulative amphotericin B deoxycholate dose administered during induction was 252.22 mg (as recorded in the medical charts). Additional antimicrobials for empiric or coinfection management included clindamycin (59.7%), trimethoprim/sulfamethoxazole (27.4%) and antituberculous therapy (12.9%).

Adverse events were frequent: AKI occurred in 59.7% (*n* = 37) and hypokalemia in 56.5% (*n* = 35). Mean hospitalization length was 14.9 days (range 3–34). Overall in-hospital mortality (mRS score 6) was 46.8% (*n* = 29). Among survivors, moderate (mRS 3) and moderately severe (mRS 4) disabilities were the most common discharge statuses (each 17.7%), followed by severe disability (mRS 5) 9.7% (*n* = 6), and mild disability (mRS 2) 6.5% (*n* = 4).

### Statistical associations

Mortality was significantly higher among patients with AKI compared with those without (35.5% vs. 11.3%; chi-square test = 5.93; *p* = 0.015) and among patients with hypokalemia (chi-square test = 5.64; *p* = 0.017). A non-significant trend toward lower mortality was observed in patients treated with daily amphotericin versus alternate-day dosing (10% vs. 45%; chi-square test = 1.08; *p* = 0.297). Patients who received combined antimicrobial regimens for suspected coinfections had numerically lower mortality (11.3% vs. 41.9%; *p* = 0.148) but this did not reach statistical significance. Regarding functional outcome, higher degrees of disability at discharge were significantly associated with the presence of hypokalemia (chi-square test = 16.66; *p* = 0.001) and with AKI (chi-square test = 13.56; *p* = 0.019); an association between amphotericin schedule and disability was observed (chi-square test = 14.78; *p* = 0.011).

## Discussion

This prospective series from a tertiary neurology referral center demonstrates that cerebral cryptococcosis in this setting predominantly affects young, rural-dwelling adults with HIV and is associated with high in-hospital mortality and substantial residual disability (5). CSF profiles and clinical presentations were typical of meningoencephalitis, but India-ink microscopy had limited sensitivity, reinforcing the importance of complementary diagnostics (cryptococcal antigen and culture) when available (6).

Treatment relied largely on amphotericin B deoxycholate plus fluconazole because flucytosine and lipid formulations were not available. The observed high frequency of AKI and hypokalemia and their strong association with mortality and worse functional outcomes underscore amphotericin toxicity as a major determinant of prognosis in resource-limited environments (7). While a numerical trend suggested better survival with daily amphotericin compared to alternate-day dosing, the difference was not statistically significant in this cohort; conversely, statistically significant associations between treatment schedule and disability were observed, possibly reflecting complex interactions among dosing, cumulative exposure, supportive care and selection bias (sicker patients may have received alternate dosing) (5, 8, 9). These findings call for cautious interpretation and indicate the need for controlled comparisons.

Compared with reports from high-income settings where mortality is lower, our observed mortality aligns with other Latin American and sub-Saharan African series, likely reflecting delays in diagnosis, high prevalence of uncontrolled HIV, and limited access to less toxic antifungals and adjunctive care (10–12). The high burden of treatment-related renal injury highlights an actionable target: structured nephroprotection bundles could reduce treatment interruptions and secondary complications (7). Operationally, a pragmatic bundle for amphotericin B deoxycholate induction could include: baseline renal function and electrolyte assessment (serum creatinine, BUN, and eGFR when available) with urine output and fluid-balance monitoring; standardized isotonic saline hydration before and after each infusion when not contraindicated; scheduled monitoring of renal function and electrolytes during induction with protocolized potassium and magnesium repletion (and bicarbonate when available); avoidance or minimization of concomitant nephrotoxins (e.g., NSAIDs, aminoglycosides, intravenous contrast) and optimization of volume status; predefined criteria for temporary interruption, dose adjustment, or regimen modification when renal function deteriorates or clinically significant electrolyte disturbances occur; and consistent documentation of cumulative amphotericin exposure and the timing of abnormalities to improve attribution and comparability across cohorts (7). Operationally, a pragmatic nephroprotection bundle for amphotericin B deoxycholate induction could include: baseline assessment of renal function and electrolytes prior to initiation; standardized isotonic saline hydration before and after each infusion when not contraindicated; scheduled monitoring of renal function and electrolytes during induction with protocolized potassium (and magnesium, when available) repletion; avoidance or minimization of concomitant nephrotoxins (e.g., NSAIDs, aminoglycosides, intravenous contrast) and optimization of volume status; predefined criteria for temporary interruption, dose adjustment, or regimen modification when renal function deteriorates or clinically significant electrolyte disturbances occur; and consistent documentation of cumulative dose and adverse events to improve continuity of care (13).

Limitations of this study include the single-center design and modest sample size, which may limit statistical power and the precision of subgroup comparisons. In addition, the cohort reflects hospitalized patients in a tertiary neurology referral center, which may over-represent more severe presentations and limits generalizability to milder or outpatient-managed disease. Outcomes were assessed only during hospitalization (in-hospital mortality and mRS at discharge), and we could not capture longer-term mortality, relapse, or post-discharge functional recovery. Also, confirmatory microbiologic diagnostics were not uniformly available: cryptococcal antigen testing and CSF culture were not systematically performed for all patients during the study period. As a result, some cases, including India-ink negative presentations, may not have had definitive microbiologic confirmation, which can introduce diagnostic misclassification. In particular, nephrotoxicity was analyzed using a binary AKI designation rather than continuous renal-function markers. Serial serum creatinine trends, BUN, and eGFR were not consistently recorded in a standardized way for all patients, preventing AKI staging and limiting assessment of nephrotoxicity severity and dose–toxicity relationships. Additionally, hypokalemia was analyzed as an in-hospital electrolyte abnormality and should not be interpreted as a stage of nephrotoxicity. Because pre-admission potassium values and standardized serial renal-function measures (e.g., serum creatinine and derived eGFR) were not consistently documented for all patients, we could not distinguish chronic from treatment-emergent hypokalemia or grade renal injury severity. Future studies in similar settings should prioritize systematic access to cryptococcal antigen testing and culture confirmation to strengthen case ascertainment and outcome comparisons (14, 15). In parallel, emerging translational platforms, including organoid-based infection models, may complement clinical studies by enabling controlled investigation of host–pathogen interactions and therapeutic screening, although such technologies remain largely inaccessible in many low-resource settings (16).

In resource-limited settings, cerebral cryptococcosis in HIV-infected adults causes high mortality and disability; prevention of amphotericin-related nephrotoxicity (hydration protocols, potassium supplementation, early detection of AKI) should be standard practice. Wider access to less nephrotoxic formulations and to flucytosine could reduce treatment-related complications and improve outcomes. Future prospective, multicenter studies should evaluate standardized nephroprotection bundles and compare amphotericin dosing schedules and formulations with respect to efficacy and safety.

## Data Availability

The datasets presented in this article are not readily available. Requests to access the datasets should be directed to fvisconti@cientifica.edu.pe.
